# Assessing maximum oxygen uptake through a motor-cognitive reactive agility test in team ball sports athletes

**DOI:** 10.3389/fspor.2026.1749132

**Published:** 2026-02-06

**Authors:** Thorben Hülsdünker, Khatija Bahdur, Laetitia Flammang, Fernando Naclerio, Yannick Sondermann, Andreas Mierau, Bettina Karsten

**Affiliations:** 1 Department of Sport, LUNEX, Differdange, Luxembourg; 2 Luxembourg Health & Sport Sciences Research Institute (LHSSRI), Differdange, Luxembourg; 3 Institute for Lifecourse Development, School of Human Sciences, Centre for Exercise Activity and Rehabilitation, University of Greenwich, London, United Kingdom; 4 Institute of Sports and Sports Science, Karlsruhe Institute of Technology, Karlsruhe, Germany; 5 Faculty of Health, Pedagogy & Social Science, Cologne University of Applied Sciences, Cologne, Germany

**Keywords:** aerobic performance, ball sport, endurance, physiological profile, reactive agility, team sport

## Abstract

**Background:**

Conventional laboratory and field tests often underestimate VO_2max_ and fail to reflect the reactive agility, multidirectional demands of team ball sports. This study examined whether a motor-cognitive Reactive Agility (RA) Test can elicit a true VO_2max_ response and serve as a sport-specific alternative for assessing VO_2max_ in team sport athletes.

**Method:**

Fifty-three team ball sports athletes performed an exhaustive incremental treadmill test and a motor-cognitive RA Test. The RA Test was performed on the SKILLCOURT and contained four all-out reactive agility runs of 150 m with an intermittent break of 30 s. VO_2max_ was determined in both tests using a portable gas analyzer. Dependent *t*-tests, Blant-Altman analysis, concordance correlation coefficient (CCC), intraclass correlation coefficient (ICC) and correlation analyses were performed.

**Results:**

The mean difference in VO_2max_ between the tests was 0.25 mL × kg^−1^ × min^−1^ (0.5%, p = 0.55) with upper and lower 95% limits of agreement at 6.02 (11%) and −5.53 (10%) mL × kg^−1^ × min^−1^, respectively. CCC (*p*_c_ = 0.94), ICC (0.943) and correlation analysis (*r* = 0.94) revealed a strong agreement and relation between VO_2max_ in the treadmill and RA Test.

**Conclusion:**

The RA Test reliably elicits a true VO_2max_ response and offers a valid and more sport-specific option when compared to laboratory treadmill assessment for measuring VO_2max_ in team ball sport athletes.

## Introduction

The maximum oxygen uptake (VO_2max_) is widely recognized as a key indicator for athlete's aerobic performance (1). In football, for instance, a high level of aerobic fitness is essential for rapid recovery between high-intensity efforts, sustaining performance during competitive matches, and covering greater total distances during the game (2, 3). Furthermore, aerobic capacity has been consistently shown to correlate with overall performance in team ball sports (4, 5), making it a fundamental component of performance assessments (6, 7).

Although aerobic endurance assessments conducted in the lab or on-court, are reliable and provide valuable information in team ball sports, they often fail to capture the full multifactorial and dynamic nature of actual match performance.

Game sports involve not only physical capabilities, but also decision-making and reactive responses in constantly changing game contexts requiring intermittent high-intensity efforts (8, 9). As a result, some researchers have questioned the relevance and ecological validity of commonly used endurance, strength and sprint performance assessments, for example, in football, and even argued that many current physical performance tests lack sufficient scientific evidence to support their use (10).

Considered the gold standard (11–13) for assessing aerobic capacity, VO_2max_ is typically measured using incremental treadmill ramp tests performed to exhaustion. However, this approach has been criticized for its low ecological validity as the movement characteristics in a treadmill ramp test (continuous linear running) do not reflect the intermittent and multidirectional activity pattern typical of team ball sports such as football (14). Therefore, the objective should be to develop a test protocol for aerobic capacity assessment that incorporates the intermittent change-of-direction (CoD) and reactive agility demands of team ball sports while still eliciting a true VO_2max_ response for direct physiological measurement.

In football alternative field-based tests, such as the Yo-Yo Intermittent Recovery (IR) tests (15), have been developed to better reflect the sport's demands. Nearly half of elite football practitioners reported assessing aerobic capacity using the YoYo-IR1 (22%) and YoYo-IR2 (24%), whereas only 15% use treadmill-based VO_2max_ assessments (6). The Yo-Yo IR test consists of repeated 2 × 20 m shuttle runs interspersed with 10-sec active recovery periods with running speed increasing from 10 km ·h^−^¹ (IR1) or 13 km·h^−^¹ (IR2) until exhaustion. VO_2max_ is estimated from the total distance covered showing moderate-to-strong correlations with treadmill-measured VO_2max_ (*r* = 0.43–0.87) (16).

The available evidence supports the use of the Yo-Yo test to estimate VO_2max_ in team ball sports; however this relationship is characterized by substantial variability, with only about 50% of the variance explained (based on an average correlation of *r* ≈ 0.7 between Yo-Yo test performance and VO_2max_ across studies) (16). Importantly, the YoYo test protocol does not allow directly measuring VO_2max_ as oxygen uptake at exhaustion is significantly lower when compared to VO_2max_ reached in a treadmill assessment (17, 18). Accordingly, while the running profile in a YoYo test better resembles the intermittent activity in team ball sports, it does not elicit a VO_2max_ response. More promising results have been reported for an agility-like test developed by Born et al. (19). Instead of 2 × 20 m linear runs with a 180 ° turn as in the YoYo test, participants performed 40 m of reactive agility (RA) runs with multidirectional CoD and intermittent breaks of 10 s to better reflect the multidirectional CoD profile and RA demands in team ball sports (20, 21). In this study VO_2max_ values were closer although still approximately 3.1% lower to those measured during treadmill-based VO_2max_ testing. Further, the correlation between VO_2max_ measured in the agility-like test and the treadmill test was comparatively low (*r* = 0.59) suggesting high variability and low agreement. From a methodological perspective it also needs to be considered that although the agility-like test enhanced the sport-specificity of endurance assessments, the use of incremental speed increases does not accurately reflect the nature of ball sports, which are characterized by frequent bouts of high- or maximal-intensity efforts (22).

Building on the findings of Erdogan et al. (2024) which demonstrated that VO_2_ values exceeding 80% of VO_2max_ can be achieved during a single 100 m RA run, an intermittent RA test incorporating multidirectional CoD movements was developed using the SKILLCOURT technology. This RA Test accounts for the motor and cognitive demands, multidirectional CoD movements and high intensity profile characteristics of team ball sports. In our laboratory, the RA Test has shown good correlations between total running time and treadmill-determined VO_2max_ [*r* = −0.800 (*r*^2^ = 0.64; 95% CI: −0.571, −0.913)] (Karsten et al. 2025—under review), which are comparable with results reported for the YoYo-test (*r* = 0.43–0.87) (16). However, it remains unclear whether VO_2max_ can truly be reached, or if the RA Test underestimates VO_2max_, as has been previously observed for the YoYo test (17, 18) and the incremental agility-like test (19).

To the best of our knowledge no endurance test protocol currently exists for team ball sport athletes that combines an intermittent, all-out RA running pattern while eliciting a VO_2max_ response comparable to that achieved in a ramp-based treadmill test. While current intermittent and more sport-specific protocols do not reach VO_2max_ and only provide estimations with limited validity, treadmill-based tests lack sport-specificity. Therefore, the present study aimed to evaluate whether a directly measured VO_2max_ can be attained during a RA test in team ball sport athletes, by comparing it to VO_2max_ values obtained from a gold-standard, ramp-like treadmill protocol. In addition, VO_2max_ was estimated from the RA test running time according to the prediction model proposed by Karsten et al. (2025—under review). Based on the available literature, we hypothesized that VO_2_ values measured or estimated in the RA Test, will not differ significantly from VO_2max_ determined via the treadmill assessment. Furthermore, we expected strong correlations and agreement between VO_2max_ values directly measured in the RA Test and treadmill test as well as between the estimated and directly measured VO_2max_ values_._ The results may provide athletes and coaches in team ball sports additional options for directly assessing VO_2max_ using a more sport-specific RA running protocol.

## Methods

### Sample size estimation

An *a priori* power analysis was conducted using G*Power (version 3.1.9.3) (23) to determine the minimum number of participants required to detect a potential difference in VO_2max_ between the treadmill and RA test. Assuming a paired-samples *t*-test, a two-tailed α level of 0.05, statistical power (1 – β) of 0.90, and a small-to-moderate effect size (*d* ≤ 0.5), the analysis indicated a minimum sample size of 44 participants (critical *t* = 2.02, *df* = 43). With 53 participants included in the final analysis, the study exceeded this requirement, achieving an actual power of 0.90 and a minimum detectable effect size of *d* = 0.41, thus ensuring sufficient power to detect meaningful differences in VO_2max_ between protocols.

### Participants and ethics

Sixty participants from the sport science student community of the university and local sport clubs were initially recruited for the study. Seven were excluded from the analysis due to missing data, failure to complete both tests, and, due to not reaching VO_2max_ during the treadmill test. The final sample contained 53 participants (27 females, 26 males) The participant characteristics are summarized in Table 1.

**Table 1:** Summary of participant characteristics across participants as well as males and females separately. Values are presented as mean (±standard deviation).

Parameter	All participants	Males	Females
Age	21.84 (4.43)	20.71 (3.88)	22.85 (4.70)
Height	174.25 (8.39)	178.38 (9.15)	170.29 (5.18)
Weight	69.62 (12.50)	71.34 (13.32)	67.95 (11.67)
BMI	22.89 (3.70)	22.30 (2.96)	23.46 (4.26)
Training hours per week	6.81 (3.13)	6.60 (3.39)	7.44 (2.24)
Years of training experience	12.12 (6.01)	10.23 (4.97)	14.17 (6.45)

Participants were trained team ball sport athletes (football, handball, volleyball, hockey, basketball) corresponding to tier 2 and tier 3 according to the classification of McKay (24). Participants had on average 12.1 (±6) years of training experience, performed 4.1 ± 1.6 training sessions per week with a weekly training load of 6.8 (±3.1) hours. Participants with muscular injuries, cardio-vascular diseases or any other limitation on the test day (e.g., sickness) were excluded. Participants were informed about the experimental protocol, and written consent was obtained prior to testing. The study was approved by the Luxembourgish national research ethics committee (Nr. 202207/01 v2.0) and conducted in accordance with the Declaration of Helsinki.

### Experimental protocol

Participants visited the lab on two occasions with at least 48 h between. To avoid influence of circadian rhythm tests were performed at the same time of the day ± 3 h. Participants were instructed to abstain from alcohol and caffeinated drinks at least 24 h prior to testing and maintain their habitual diet. Moreover, there should be no intense training on the day before the lab visit.

On day 1, participants completed an exhaustive treadmill VO_2max_ test followed by a familiarization trial with the RA Test on the SKILLCOURT to minimize potential learning effects. On day 2, they performed the RA Test. During all tests, gas exchange was continuously measured breath-by-breath using a validated mobile MetaMax 3B analyzer (CORTEX Biophysik GmbH, Leipzig, Germany) (25). The gas analysis system was calibrated according to the manufacturer guidelines using reference gas and ambient air calibration as well as flow sensor volume calibration with a 3 L calibration syringe. Heart rate (HR) was continuously measured using a H10 sensor (Polar Elektro, Kempele, Finland). Rate of perceived exertion (RPE; 6–20) was obtained according to Borg's scale. Blood lactate samples were taken at the earlobe and analyzed using a Biosen C-Line lactate analyzer (EKF-diagnostic GmbH, Barleben, Germany).

### Treadmill ramp test

**Figure 1:**
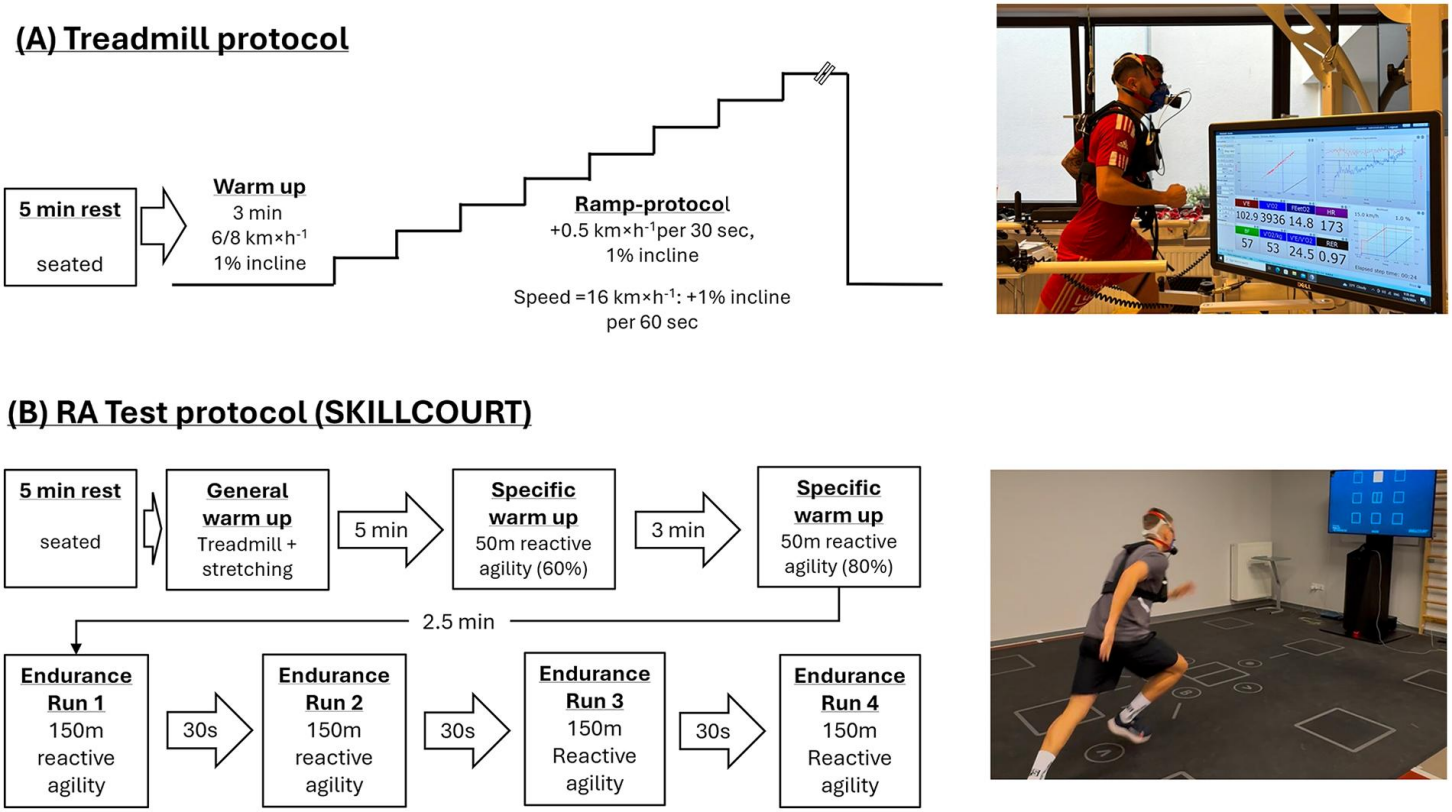
Testing protocols for the **(A)** the treadmill ramp test and **(B)** the RA test.

The ramp-like incremental test was performed on a treadmill (h/p/cosmos®, Pulsar®, Nussdorf, Germany). To account for differences in performance, participants started either at 6 km × h^−1^ or 8 km × h^−1^ with a 1% incline (26). The decision was taken based on training experience, number of weekly training sessions, training load, previous performance tests (if available) and personal rating of performance status. The protocol for the treadmill test is illustrated in Figure 1A. Participants warmed up for 3 min at the starting velocity. This was followed by a speed increase of 0.5 km × h^−1^ every 30 s up to a velocity of 16 km × h^−1^. Afterwards, inclination was increased by 1% per minute. Participants were verbally encouraged throughout the test. RPE and lactate were determined prior to and immediately after the test. VO_2_ and HR were continuously recorded. The average test duration was 10.7 (±2.2) min. A 30s moving average was applied to the raw data and VO_2max_ was defined as the highest VO_2_ value. VO_2max_ was considered as valid if the VO_2_ increase during the last minute did not exceed 150 mL indicating a levelling-off (27). Alternatively, two of the four criteria must be met. 1) RER≥1.1, 2) blood lactate concentration ≥ 8mmol × l^−1^, 3) HR ≥ 95% of maximum HR (220-age) or 4) RPE ≥ 18 (19, 28). Participants, neither reaching the primary (levelling-off) nor secondary criteria, were excluded from the analysis.

### Reactive agility test

On test day 2, participants performed the RA Test on the SKILLCOURT (Skillcourt GmbH, Schweinfurt, Germany). The test contains four all-out 150 m reactive agility runs with 30 s of rest in between runs that were performed on a 4 × 4 m court. The test protocol for the RA Test is illustrated in Figure 1B. Participants started with a warm-up comprising 3 min of moderate, self-paced treadmill running followed by 5 min of stretching. Afterwards, two RA runs of 50 m were performed on the SKILLCOURT. Participants were asked to perform the runs at 60% and 80% of their individual maximum performance. The intensities were chosen to increase the physiological demands following the general warm-up and prepare participants for the maximum intensity all-out RA runs. After 2.5 min recovery, the first run started. Participants had to run to one out of eight target fields as indicated on the device screen. Once a field was reached the next showed up. The sequence was randomized, and distance was automatically calculated by the system using a LiDAR (Light Detection and Ranging). Participants were instructed to perform each run as fast as possible and received verbal encouragement throughout the test. To ensure consistency in effort and time, in the event of a RA error run, the test proceeded, and participants continued to the next target field. As for the treadmill test, RPE and lactate were determined prior to and immediately after the test. VO_2_ and HR were continuously recorded during both tests. The test time for the RA Test was 8.2 (±0.5) min (6.2 min running time + 4 × 30 s break). As for the treadmill test, a 30s moving average was applied to the raw data and the highest VO_2_ was considered as VO_2max_.

In addition to its direct measurement, VO_2max_ was estimated through an equation (Equation 1) established from a previous study, which investigated the relationship between total running time in the RA Test and treadmill-based VO_2max_ (Karsten et al. 2025—under review).

$$\mathrm{estimated}\;VO_{2\max}=-0.1681\;\times \mathrm{RA}\;\mathrm{Run}\;\mathrm{Total}\;\mathrm{Time}\;\;(s)\;+115.65$$

All metrics for the treadmill and RA Test are presented in Table 2.

**Table 2:** Summary of parameters derived from the treadmill VO_2max_ test and RA test. Values are presented as mean (±standard deviation).

**Parameter**	All participants	Males	Females
Treadmill ramp test			
Test duration (min)	10.72 (2.18)	11.47 (1.84)	9.93 (2.26)
Peak oxygen uptake (mL × kg^−1^ × min^−1^)	53.02 (8.69)	57.31 (7.35)	48.89 (7.94)
Heart rate pre (bpm)	73.96 (14.57)	72.04 (13.00)	75.81 (15.96)
Lactate pre (mmol × l^−1^)	1.01 (0.26)	1.05 (0.26)	0.98 (0.25)
RPE pre (6–20)	6.16 (0.47)	6.00 (0.00)	6.33 (0.64)
Heart rate post (bpm)	191.82 (7.22)	192.61 (7.10)	191.00 (7.45)
Lactate post (mmol × l^−1^)	8.31 (2.16)	8.15 (1.93)	8.46 (2.39)
RPE post (6–20)	18.11 (1.60)	18.58 (1.23)	17.67 (2.07)
RA Test			
Test duration (min)	8.28 (0.57)	8.00 (0.44)	8.57 (0.55)
Peak oxygen uptake (mL × kg^−1^ × min^−1^)	53.02 (8.69)	56.92 (7.83)	48.78 (7.75)
Heart rate pre (bpm)	72.60 (10.95)	70.61 (11.06)	74.29 (10.78)
Lactate pre (mmol × l^−1^)	1.10 (0.26)	1.09 (0.25)	1.10 (0.27)
RPE pre (6–20)	6.15 (0.35)	6.00 (0.00)	6.30 (0.47)
Heart rate post (bpm)	190.00 (7.16)	188.91 (7.58)	191.05 (6.76)
Lactate post (mmol × l^−1^)	9.04 (2.72)	9.17 (2.35)	8.90 (3.10)
RPE post (6–20)	19.45 (0.79)	19.42 (0.78)	19.48 (0.82)

### Statistical analysis

Data was analyzed in JASP (version 0.19.0.3) and SPSS (version 29.0.2.0). Shapiro–Wilk tests were used to test for normal distribution and non-parametric tests were used in case of normal distribution violation. Control analyses tested for differences in resting state HR, LA, RPE and test duration.

Agreement between treadmill VO_2max_ and RA Test VO_2max_ was assessed using Limits of Agreement (LoA) according to Bland and Altman (29). Lin's concordance correlation coefficient (CCC) for paired measurements (30) and Pearson correlation coefficient were used for correlation analysis between VO_2max_ measured in the RA Test and treadmill VO_2max_. A dependent t-test was applied to test for significant differences in measured VO_2max_ between the treadmill and RA Test. The intraclass correlation coefficient (ICC) was calculated based on a two-way mixed effects model with single measurement and absolute agreement (31) according to the classification of McGraw and Wong (32) to determine the degree of similarity in VO_2max_ between the treadmill and RA Test. The same set of analyses was performed to compare the treadmill VO_2max_ to the predicted VO_2max_ values based on the overall running time in the RA Test. To account for potential sex differences, all analyses were also carried out separately for male and female participants.

Effect sizes were considered small (*d* = 0.2, *r* = 0.1), medium (*d* = 0.5, *r* = 0.3) or large (*d* = 0.8, *r* = 0.5). CCC was considered nearly perfect [precision(p_c_) > 0.99], substantial (p_c_ > 0.95), moderate (p_c_ > 0.9) or poor (p_c_ ≤ 0.9). The significance threshold was set to *p* < 0.05.

## Results

Control analyses did not reveal differences in physiological measures (HR, La, RPE) between the two test days (*p* ≥ 0.122). With a duration of 8.2 (±0.6) minutes, the RA Test was significantly shorter when compared to the treadmill test (10.7 ± 2.2; *p* < 0.001).

### Measured VO_2max_

**Figure 2:**
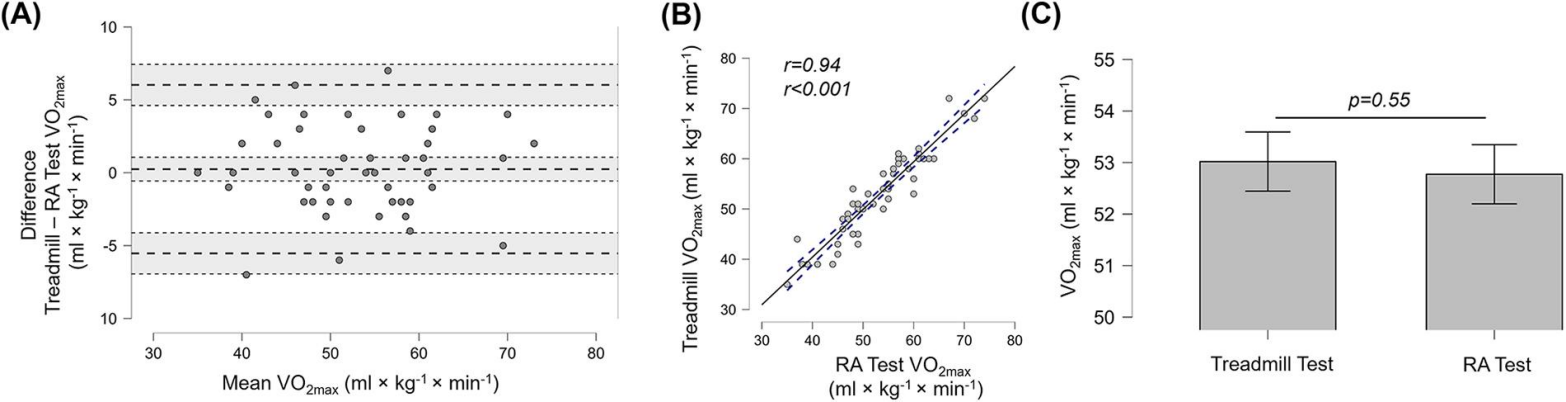
**(A)** Bland-Altman plots indicating difference in means and the 95% limits of agreement, **(B)** linear relationship between VO_2max_ measured in the treadmill test and RA test and **(C)** the T-test results comparing VO_2max_ measured during treadmill test and the RA test for the whole group (*n* = 53). Error bars indicate 95% confidence intervals.

No significant difference was found between VO_2max_ values obtained from the RA Test and the treadmill protocol (*t* = 0.606, *p* = 0.547, *d* = 0.083). Bland–Altman analysis showed a mean bias of 0.25 mL·kg^−^¹·min^−^¹**,** with 95% LoA of +6.02 (∼11%) and −5.53 mL·kg^−^¹·min^−^¹ (∼10%)**,** indicating a negligible underestimation (<0.5%) of VO_2max_ in the RA Test. The Lin's concordance correlation coefficient (*ρ*c = 0.94) demonstrated moderate agreement between methods. A very strong Pearson correlation was observed (*r* = 0.94, *p* < 0.001), and the intraclass correlation coefficient (ICC = 0.943; 95% CI 0.907–0.966) confirmed excellent agreement between treadmill and RA Test VO_2max_ measurements. The findings from the overall (whole-group) analysis are depicted in Figure 2.

### Estimated VO_2max_

**Figure 3:**
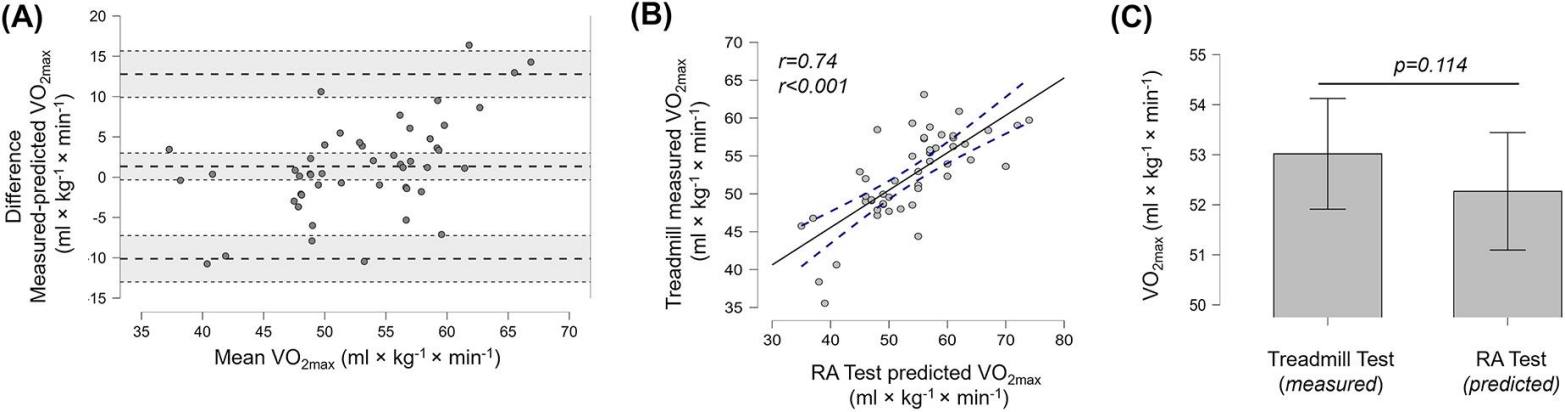
**(A)** Bland-Altman plots indicating difference in means and the 95% limits of agreement, **(B)** linear relationship between VO_2max_ measured in the treadmill test and predicted from the RA test and **(C)** the T-test results comparing VO_2max_ measured during treadmill test and predicted from the RA test for the whole group (*n* = 53). Error bars indicate 95% confidence intervals.

LoA analysis between estimated and measured VO_2max_ on the treadmill revealed a difference of 1.33 mL × kg^−1 ^ ×  min^−1^ (3.9%) with upper and lower 95% LoA of 12.8 (24%) and −10.1 (19%) mL × kg^−1^ ×  min^−1^, respectively. There was no significant difference between VO2_max_ measured on the treadmill and predicted form the running time in the RA Test (*t* = 1.610, *p* = 0.114, *d* = 0.228). Strong correlations were observed between the estimated VO_2max_ in the RA Test and the measured treadmill VO_2max_ (*r* = 0.74, *p* < 0.001). However, with a coefficient of concordance of p_c_ = 0.736, the agreement was rather poor. Also, the ICC of 0.674 indicated only moderate similarity between measured treadmill VO_2max_ and estimated values from the RA Test. Results are presented in Figure 3.

### Male vs. female team ball sport athletes

Results for the subgroups of male and female athletes are summarized in Table 3. The findings from the overall analyses were consistent across subgroups, particularly for the directly measured VO_2max_ in the RA test. While for the predicted VO_2max_, there was a significant difference between the treadmill and RA test only for the male group (*t* = 2.457, *p* = 0.022, *d* = 0.491) and ICC and CCC where lower in absolute numbers in the male when compared to the female participants, effect sizes for the t-test as well as ICC and CCC values were not significantly different (*p* ≥ 0.171).

**Table 3:** Summary of statistical results for all participants as well as males and females separately.

Test	All participants (*n* = 53)	Males (*n* = 26)	Females (*n* = 27)
Measured VO_2max_			
Bland-Altman statistics	Mean: 0.245	Mean: 0.385	Mean: 0.111
	95%: −5.532 to 6.023	95%: −6.082 to 6.851	95%: −5.027 to 5.249
*T*-test	*T* = 0.606, *P* = 0.547, *D* = 0.083	*T* = 0.594, *P* = 0.558, *D* = 0.117	*T* = 0.220, *P* = 0.827, D = 0.042
Correlation	R = 0.943, *P* < 0.001	R = 0.907, *P* < 0.001	R = 0.969, *P* < 0.001
Intraclass correlation coefficient (ICC)	0.943	0.913	0.944
Concordance correlation coefficient (CCC)	*ρ*c = 0.94	*ρ*c = 0.908	*ρ*c = 0.945
Predicted VO_2max_			
Bland-Altman statistics	Mean: 1.330	Mean: 2.786	Mean: −0.126
	95%: −10.122 to 12.783	95%: −8.327 to 13.900	95%: −11.409 to 11.157
*T*-test	*T* = 1.610, *P* = 0.114, *D* = 0.228	***T*** ***=*** ***2.457, P*** ***=*** ***0.022, D*** ***=*** ***0.491***	*T* = −0.109, *P* = 0.914, *D* = −0.022
Correlation	*R* = 0.737, *P* < 0.001	*R* = 0.544, *P* = 0.005	*R* = 0.710, *P* < 0.001
Intraclass correlation coefficient (ICC)	0.674	0.477	0.659
Concordance correlation coefficient (CCC)	pc = 0.736	pc = 0.544	ρc = 0.710

Bold values indicate significant results.

## Discussion

This study evaluated if repeated reactive agility sprints can elicit a VO_2max_ response in team ball sport athletes. As the results were not statistically different across parameters between sexes, the following discussion focuses on the group as a whole (*n* = 53). The observed results when directly measuring VO_2_ using a gas analyzer revealed close to identical average VO_2max_ values in both tests (<0.5% difference) and a strong relation and agreement between the treadmill and RA Test. These findings support the use of RA Test protocols to elicit VO_2max_. The prediction based on overall running time in the RA Test only provided limited validity for VO_2max_ estimation.

### Measured maximal oxygen uptake (VO_2max_)

With a mean difference of 0.25 mL × kg^−1^ ×  min^−1^, corresponding to about 0.5%, there was a strong agreement in the measured VO_2max_ between the two tests. The agreement achieved in the RA Test was substantially stronger than those reported by Martinez-Luganas and Hartmann (18) who found the Yo-Yo IR1 underestimated treadmill VO_2max_ by 9.4% (95% LoA: −20% to +1.4%) in female football players. Similarly Castagna et al. (17) observed a 2.67 mL·kg^−^¹·min^−^¹ (∼5%) underestimation in male youth players, with LoA of −14 to +8.6 mL·kg^−^¹·min^−^¹ (−26% to +16%), roughly twice as wide as in the RA Test. Interestingly, the underestimation of VO_2max_ was substantially smaller (1.7 mL × kg^−1^ ×  min^−1^, 3.1%) in the YoYo-IR2 test as reported by Born et al. (19). The same applied to the incremental test on the SpeedCourt incorporating a RA component (1.7 mL × kg^−1^ ×  min^−1^, 3.1%). LoA were substantially larger when compared to the RA Test with about −9–9 mL × kg^−1^ × min^−1^ (−16% to +16%) for the YoYo-IR2 and −6 to +10 mL × kg^−1^ ×  min^−1^ (−10.8% to +18%) for the incremental RA test on the SpeedCourt.

Although the LoA in the present study (+11% and −10%) may still be considerable wide, this degree of variability falls within the known biological and technical error of VO_2max_ testing. Katch et al. (33) reported a total error of 5.6% across repeated maximal tests. An additional 2% error is attributable to gas-analyser variability (34). Hence, the combined error margin (∼7%) supports the conclusion that the RA Test demonstrates good validity for eliciting VO_2max_.

The correlation between VO_2max_ measured on the treadmill and in the RA Test (*r* = 0.94) was very high and significantly stronger when compared to the correlation reported between treadmill VO_2max_ and the YoYo-IR1 test by Martinez-Luganas and Hartmann (18) (*r* = 0.94 vs. *r* = 0.83, *p* = 0.039). The study by Castagna et al. (17) observed an even lower correlation between treadmill and YoYo-IR1 VO_2peak_ of *r* = 0.65. Interestingly, while the agility-like test on the SpeedCourt was closest in reaching the treadmill VO_2max_, the correlation between SpeedCourt and treadmill VO_2max_ was comparatively low (*r* = 0.59) (19). When considering the strong correlation between the treadmill and RA Test together with the very high concordance correlation coefficient (p_c_ = 0.94) and ICC (0.94), these findings confirm excellent agreement between VO_2max_ values measured during the treadmill and RA Test.

In contrast to other field-based protocols, the motor-cognitive RA Test reliably elicits VO_2max_ while replicating the reactive multidirectional, and cognitive demands of team ball sports. Accordingly, the RA Test provides a valid, sport-specific, and practically applicable option for assessing aerobic capacity in team ball sport athletes.

### Estimated VO_2max_

The predicted VO_2max_ based on the overall running time in the RA Test also did not indicate a significant difference from the directly measured VO_2max_ on the treadmill. The estimation in the RA Test demonstrated greater precision than previously reported for the YoYo test, where the VO_2max_ predicted using the Bangsbo et al. (15) equation underestimated the treadmill-measured VO_2max_ by 17.8% in female football players (18). Comparable values were reported by Michailidis et al. (35) for male football players where VO_2max_ was underestimated by 14% in the YoYo-IR1. The largest difference was observed by Kramer et al. (36) with an underestimation of 30% when comparing estimated VO_2max_ to measured VO_2max_ in a YoYo-IR1 test. Although the prediction of VO_2max_ from the RA Test was on average accurate, the LoA were substantially wider than those observed for directly measured VO_2max_ and comparable to previous findings for the YoYo-test (18). Considering the wider LoA between 24% and −19% together with the concordance correlation coefficient of p_c_ = 0.736 and the ICC of 0.68, the estimated VO_2max_ from the RA Test should therefore be interpreted as an approximate indicator rather than a precise prediction of VO_2max_. This interpretation is further supported by the correlation analysis which revealed an association of 0.737, similar to the range typically reported for the YoYo test [*r* = 0.43 and *r* = 0.87 (16)]. Given the RA Test's reactive and multidirectional characteristics, it is plausible that CoD ability and anaerobic energy contributions influence running performance and, consequently, reduced the strength of the correlation. In fact, CoD ability does depend on strength, power and technique which may substantially vary between participants independent of aerobic capacity (37). Moreover, there was only a moderate correlation (*r* = −0.346) between VO_2max_ and overall running time in an 8 × 40 m sprint protocol (38). Therefore, although the running distance was longer in this study (4 × 150 m), a substantial contribution of the anaerobic energy metabolism to the overall running time in the RA Test can be assumed.

### Practical application

The motor-cognitive RA Test was specifically designed for team ball sport athletes to provide a higher stimulus-correspondence (through external visual cue) and task correspondence (through all-out multidirectional CoD) compared to existing field-based endurance tests. VO_2max_ values closely align with those obtained from treadmill testing and they provide a higher precision and accuracy in obtaining VO_2max_ when compared to established field-based endurance tests. The RA test can therefore serve as an option and alternative for athletes in team ball sports to determine aerobic capacity in a more sport-specific setting. As such, the RA test may qualify as a performance test for evaluating training programmes or athlete selection.

With a test duration of 8.2 ± 0.6 min, the RA Test is more time efficient than the treadmill testing (10.7 ± 2.2 min) and comparable in length to the YoYo test (18). Importantly, 90% of the participants reached their VO_2max_ within the first or second 150-m run interval, suggesting the potential to shorten the protocol to two intervals of 2 × 150-m in future applications. However, as the RA Test is an all-out test, a 5 min warm-up should be considered. Still, the total testing time was shorter when compared to the incremental agility-like test reported by Born et al. (19) (∼18–19 min). Finally, due to distance measurement by the LiDAR and automatic data processing, the test provides a high objectivity, and it is less staff-intensive when compared to e.g., a YoYo-test, although only one athlete can be tested at a time. The estimation of VO_2max_ values based on overall running time may be useful for coaches and athletes when gas analysis is not available but should be interpreted with caution. In this context the same limitations apply to the RA Test as to commonly used field-based VO_2max_ tests.

### Limitations and future directions

While the study confirms that the RA Test can obtain a VO_2max_ response, reliability has not been addressed and needs to be confirmed in future research. To support generalizability across performance levels and disciplines, additional studies with different athlete populations are warranted. Further, oxygen kinetics were not considered and should be analyzed to provide an explanation for the higher validity when compared to many existing field-based tests. Future research should also evaluate whether performance in the RA Test is related to physiological on-court performance (e.g., running distance, number of sprints, etc.) as previously indicated for the YoYo-test (15, 39, 40). Finally, although this study employed SKILLCOURT technology to measure and estimate VO_2max_, similar RA test protocols could potentially be implemented using other technologies (e.g., reaction lights), which warrants evaluation in future research.

## Conclusions

The present study demonstrates that the motor-cognitive RA Test performed on the SKILLCOURT reliably elicits a true VO_2max_ response in team ball sport athletes. VO_2max_ values showed no significant difference and a very strong agreement with those obtained from laboratory treadmill testing. While direct gas analysis remains the preferred method for precise measurement, VO_2max_ estimation from the RA Test provides a practical alternative albeit with greater variability and reduced predictive accuracy. Overall, the RA Test is a valid, objective, and sport-specific tool for assessing VO_2max_ in team ball sports.

## Data Availability

The raw data supporting the conclusions of this article will be made available by the authors, without undue reservation.
